# AVaTER: Fusing Audio, Visual, and Textual Modalities Using Cross-Modal Attention for Emotion Recognition

**DOI:** 10.3390/s24185862

**Published:** 2024-09-10

**Authors:** Avishek Das, Moumita Sen Sarma, Mohammed Moshiul Hoque, Nazmul Siddique, M. Ali Akber Dewan

**Affiliations:** 1 Department of Computer Science and Engineering, Chittagong University of Engineering and Technology, Chittagong 4349, Bangladesh; avishek@cuet.ac.bd (A.D.); moumita@cuet.ac.bd (M.S.S.); 2 School of Computing, Engineering and Intelligent Systems, Ulster University, Belfast BT15 1AP, UK; 3 School of Computing and Information Systems, Faculty of Science and Technology, Athabasca University, Athabasca, AB T9S 3A3, Canada; adewan@athabascau.ca

**Keywords:** multimodal emotion recognition, natural language processing, multimodal dataset, cross-modal attention, transformers

## Abstract

Multimodal emotion classification (MEC) involves analyzing and identifying human emotions by integrating data from multiple sources, such as audio, video, and text. This approach leverages the complementary strengths of each modality to enhance the accuracy and robustness of emotion recognition systems. However, one significant challenge is effectively integrating these diverse data sources, each with unique characteristics and levels of noise. Additionally, the scarcity of large, annotated multimodal datasets in Bangla limits the training and evaluation of models. In this work, we unveiled a pioneering multimodal Bangla dataset, MAViT-Bangla (Multimodal Audio Video Text Bangla dataset). This dataset, comprising 1002 samples across audio, video, and text modalities, is a unique resource for emotion recognition studies in the Bangla language. It features emotional categories such as anger, fear, joy, and sadness, providing a comprehensive platform for research. Additionally, we developed a framework for audio, video and textual emotion recognition (i.e., AVaTER) that employs a cross-modal attention mechanism among unimodal features. This mechanism fosters the interaction and fusion of features from different modalities, enhancing the model’s ability to capture nuanced emotional cues. The effectiveness of this approach was demonstrated by achieving an F1-score of 0.64, a significant improvement over unimodal methods.

## 1. Introduction

Emotion classification is pivotal for advancing human–computer interaction, making technological applications more intuitive, responsive, and effective. As digital technologies become increasingly integrated into our daily lives, from virtual assistants on smartphones to customer service chatbots and mental health monitoring apps, recognizing and responding to human emotions accurately becomes crucial. It enables these systems to adjust their responses based on emotional cues, creating personalized experiences and facilitating better healthcare, education, and entertainment decision-making. Emotion classification enables systems to bridge the communication gap between humans and machines. By fostering more natural interactions that mirror human-to-human exchanges, technology becomes more accessible and beneficial to diverse global populations. For example, a virtual assistant who understands when a user is frustrated can respond empathetically and helpfully, improving user satisfaction and engagement.

Various techniques have been developed for emotion classification, encompassing both unimodal and multimodal approaches. Unimodal emotion classification refers to analyzing and interpreting human emotions based on data from a single source or modality. This approach uses just one type of input—text, audio, or visual data—to determine emotional states. For instance, a text-based system might analyze the words and phrases used in a social media post to identify the user’s emotional state. On the other hand, MEC combines data from multiple sources such as text, audio, and visual inputs to analyze and predict emotions. This approach is more robust and can interpret complex emotions more effectively. For example, in a video call, a multimodal system could analyze the spoken words (text), the tone of voice (audio), and facial expressions (visual) to gain a comprehensive understanding of the user’s emotional state. This comprehensive analysis is particularly beneficial in applications that require deep understanding and insight, such as therapeutic settings or advanced customer service platforms.

In this work, we aim to push the boundaries of emotion classification by developing a computational system capable of identifying four principal categories of emotion: joy, sadness, fear, and anger. Our approach utilizes a multimodal methodology, integrating audio, video, and text inputs to achieve a more nuanced and accurate classification of emotions. This multimodal approach allows the system to capture emotional expressions, often conveyed through verbal and non-verbal cues. One of our key motivations is to bridge the resource gap for Bengali-specific datasets used in multimodal emotion analysis. There is a significant need for such datasets, which hampers the development of emotion recognition systems tailored to the Bengali-speaking population. By contributing and expanding these datasets, we aim to support the development of more effective and culturally relevant emotion classification systems for the Bengali language. The specific contributions of this work are as follows:

- Development of MAViTE-Bangla, a multimodal Bangla emotion dataset containing 1002 multimodal samples labeled into four classes: anger, fear, joy, and sadness.
- Development of a pairwise cross-attention-based multimodal framework for effective emotion recognition and exploitation of several feature extraction and fusion methods to utilize multimodal features for MEC.
- Analysis of the classification outcomes of the proposed method with a detailed investigation of the misclassification of samples

This study aims to advance the technical capabilities of emotion classification systems and ensure that these advancements are inclusive and applicable to a broader range of languages and cultural contexts. We aim to create technologically advanced and socially impactful systems by integrating multiple modalities and developing Bengali-specific resources, ultimately enhancing human–computer interactions across different cultures and languages.

## 2. Related Work

Multimodal emotion classification has evolved significantly, driven by advancements in machine learning and an increased understanding of how emotions are conveyed through multiple channels. Early research primarily focused on unimodal approaches, analyzing data types like text, audio, or visual cues. Single modalities complement each other, so combining them offers a richer, more contextual view, enhancing their generalization ability [1]. Most multimodal emotion classification research uses English and other widely known European languages. However, there is a growing desire to broaden this research to include underrepresented languages like Bengali, which presents unique opportunities and problems for furthering this field of study. This section provides and overview of the literature on unimodal and multimodal emotion categorization.

### 2.1. Unimodal-Based Emotion Recognition

Haque et al. [2] proposed a CNN-LSTM deep learning technique to classify four sentiment classes: sexual, religious, political, and acceptable. They used a dataset consisting of 42,036 labeled Facebook comments and a web application developed for real-time sentiment prediction. This model achieved 85.8% accuracy, but the authors did not consider transformer-based models that might perform better. To categorize nine kinds of emotions, Islam et al. [3] introduced EmoNaBa (Bangla language corpus), and a hybrid model was presented using transformers and lexicon features based on this dataset [4]. However, in this instance, the model’s effectiveness depends on having an extensive vocabulary of emotions, which calls for constant updates as new words and dialects appear. Three transformer models, m-BERT, BanglaBERT, and XLM-R, were assessed by Das et al. [5] utilizing a variety of DL and ML models on a corpus BEmoC [6]. This work obtained the highest weighted F1-score of 69.73% with XLM-R. Rahman et al. [7] developed a dynamic strategy leveraging the Word2Vec model, employing both skip-gram and continuous bag of words (CBOW) techniques, focusing on three emotion classes: happy, angry, and excited. This work may not capture the full range of human emotions in the text due to the lower number of classes. In contrast, the authors of [8] utilized various ML and neural network models to classify emotions from Bengali song lyrics, achieving moderate accuracy in multi-class classification. Employing an ensemble technique with CNN, GRU, and BiLSTM, Parvin et al. [9] classified six emotion categories from a new corpus containing 9000 annotated texts.

Regarding speech emotion detection, Sultana et al. [10] created a novel architecture called DCTFB, fusing BiLSTM with deep convolutional neural networks (DCNN) that classify emotions from Bengali and English speech along with song corpus. However, the model’s generalizability may be impacted by the imbalanced speaker gender and emotion classes in the RAVDESS dataset employed in this work. Similarly, in [11], three kinds of emotions (angry, happy, and neutral) were classified in Bengali speech corpus with ML methods using MFCCs (Mel-Frequency Cepstral Coefficients) and LPCs (Linear Prediction Coefficients) features extracted from audio signals. Conversely, five pre-trained deep learning models were used in [12] to categorize hateful and non-hateful emotions in a new dataset of 3000 hand-labeled Bengali memes. However, this work should have considered the multimodality of memes, where images and text jointly convey the whole meaning of the content.

### 2.2. Multimodal-Based Emotion Recognition

Regarding MEC, Ghosh et al. [13] created an emotion corpus called MELD, which includes seven classes and combines visual, textual, and audio modalities. They also evaluated the performance of several baseline models on this dataset. However, misclassification occurred when subtle emotions such as disgust and fear were classified. Hu et al. [14] presented a unified framework by merging multimodal sentiment analysis (MSA) and emotion recognition by fusing syntactic and semantic levels across the modalities on four public benchmark datasets, including MELD. Similarly, a multimodal feature fusion method was proposed in [15] to address unbalanced sample data in social network public opinion analysis. It utilized text and speech emotion features, introduced the MA2PE speech feature retrieval method, and addressed sample disequilibrium through data processing techniques with the IEMOCAP and MELD datasets. Additionally, Hossain et al. [16] proposed a model that effectively integrated different specialized architectures for each modality: CNN-LSTM for processing audio features, Inception-ResNet-v2 for extracting video features, and Word2Vec for handling text features. This comprehensive approach enabled the model to recognize four distinct emotion categories from the IEMO-CAP dataset. Similarly, the authors in [17] employed a combination of advanced techniques, using BERT for text features, wav2vec2.0 for audio features, and videoMAE for video features. They used an early fusion strategy and SVM classification to enhance the emotion recognition accuracy. However, the authors of [18] introduced an attention-based multimodal emotion detection system that fuses facial and speech features extracted by independent encoders. The system uses convolutional neural networks (CNNs) to process these features and employs an attention mechanism to focus on the most informative parts evaluated on IEMOCAP and CMU-MOSEI datasets. Apart from multimodal emotion and sentiment recognition, to detect the risk of depression, a study in [19] employed the Audio, Video, and Text Fusion-Three Branch Network (AVTF-TBN), integrating three main networks: an audio feature extractor using a convolutional neural network (CNN), a video feature extractor using a 3D CNN, and a text feature extractor using a BiLSTM network.

Regarding the Bengali language, the authors in [20] applied feature fusion and decision fusion techniques between textual and visual modalities to classify emotions in Bangla social media content despite a significantly imbalanced dataset. Hossain et al. [21] introduced a multimodal method to classify hateful memes using the newly created MUTE dataset, including Bengali and code-mixed captions, leveraging visual and textual modalities. Similarly, the researchers in [22] employed the Multimodal Attentive Fusion (MAF) model to identify five categories of aggression in a Bengali meme dataset by integrating visual and textual features. In Bengali, no research is available to date that combines audio, video, and text modalities for emotion classification using a multimodal approach. This comprehensive integration is the central focus of this work.

## 3. Dataset Description

In this research, we have created a comprehensive Bengali multimodal emotion recognition dataset, which we have named MAViTE-Bangla. This dataset comprises 1002 samples, each encompassing audio, video, and text modalities, providing a rich, multimodal resource for emotion recognition studies. The MAViTE-Bangla dataset is categorized into four distinct emotional classes, anger, fear, joy, and sadness, ensuring a diverse representation of emotional expressions.

### 3.1. Data Collection

**Figure 1:**
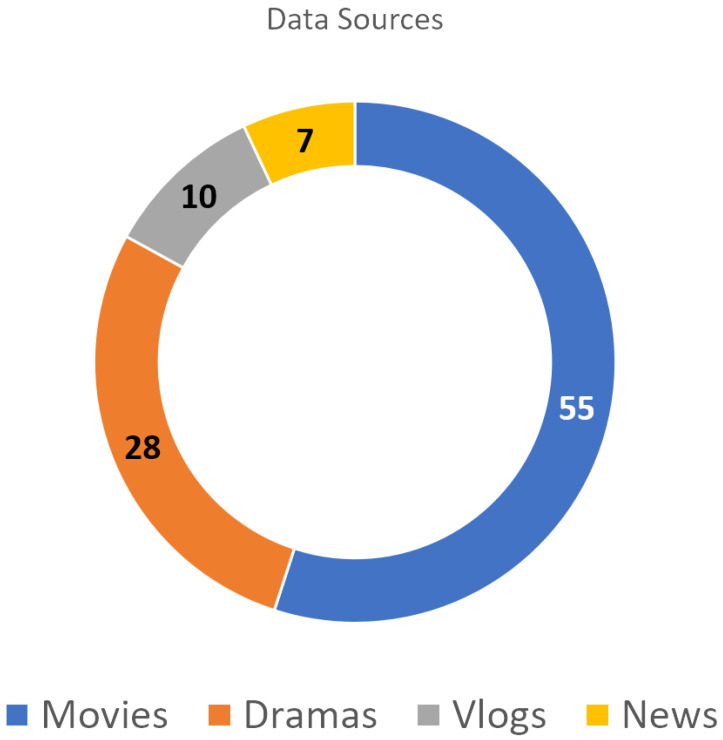
Distribution of data according to sources.

This work manually collected YouTube videos from multiple domains, such as movies, dramas, and video blogs (vlogs). This was performed to ensure that our dataset included many emotions and scenarios. Figure 1 shows a detailed breakdown of the domains represented in the dataset.

**Figure 2:**
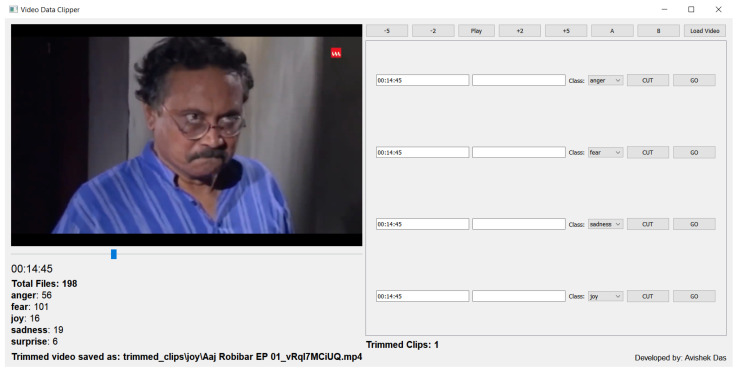
Snapshot of the data annotation tool.

We developed a data annotation tool in Python 3.10.9 using the PyQT framework. Figure 2 shows a snapshot of the tool. The main features of this tool are video loading, frame range selection, annotation, and saving. It also shows a total number of files collected in each class. In addition, forwarding/rewinding by 2/5 s is also provided for catching a particular moment. The annotation tool is available at https://github.com/avishek-018/AVaTER (accessed on 20 February 2024).

### 3.2. Data Statistics

The dataset has been systematically divided into training, validation, and test sets to facilitate practical training and evaluation of emotion recognition models. The specific distribution of samples across these sets is detailed in Table 1. By developing MAViTE-Bangla, we address the lack of multimodal emotion recognition resources for Bengali, enhancing research and aiding in creating models that accurately interpret emotions from audio, video, and text.

Table 2 presents the detailed statistics for the audio dataset, focusing on four emotional classes: anger, fear, joy, and sadness. For anger, the minimum duration was 1.022 s, while the maximum duration reached 7.002 s, with a mean duration of 3.210 s. The cumulative duration for anger samples was recorded at 950.071 s. Fear exhibited a broader range of durations, with a minimum of 2.001 s and a maximum of 14.008 s. This resulted in a higher mean duration of 5.491 s, and the total duration for fear samples was 549.061 s. On the other hand, joy had the shortest minimum duration among the emotions at 0.952 s and a maximum duration of 9.015 s. The mean duration for joy was 2.937 s, contributing to a total duration of 757.709 s. Moreover, sadness had a minimum duration of 1.207 s and a maximum of 7.012 s, with a mean duration of 3.391 s. It also had the highest total duration among the emotional classes, amounting to 986.822 s.

In the analysis of the video dataset, key metrics crucial for video classification tasks were examined and are presented in Table 3. The frame rate across all emotional classes reached a maximum of 30.0 frames per second (fps), a standard for high-quality video playback, ensuring smooth motion representation. The minimum frame rates observed were slightly lower, ranging from 23.98 to 24.00 fps, which still maintained acceptable video quality for classification tasks. The resolution analysis revealed considerable variation within the dataset. A resolution of 2560×1440 pixels, the highest observed, was found in the ‘fear’ class, indicative of high-definition video quality. High-definition videos up to 1920×1080 pixels were also in the ‘anger’, ‘joy’, and ‘sadness’ classes. However, due to resource constraints, some videos had lower resolutions: 208×210 pixels for ‘anger’, 450×360 pixels for ‘fear’, 206×174 pixels for ‘joy’, and 176×162 pixels for ‘sadness’. These lower-resolution data was collected due to resource constraints during data acquisition, highlighting the balance between resource availability and data quality. Despite these lower resolutions, crucial visual information necessary for effective classification is retained in the dataset.

The textual dataset for this study exhibits significant variations across the emotional classes, as detailed in Table 4. The joy class stands out with the highest lexical diversity, scoring 0.537 and an average sentence length of 33.685 words. This indicates a varied vocabulary and longer sentences within this class. In contrast, the sadness class features the most extended average sentence length at 63.581 words but has the lowest lexical diversity, with a score of 0.464. The anger class is notable for its high total word count, comprising 2395 words, whereas the fear class has the fewest words, totaling 996, and the least number of sentences, with only 34. These statistics highlight the diverse linguistic patterns within the dataset, which are crucial for developing emotion recognition models. The variations in sentence length and lexical diversity across the emotional classes provide a rich foundation for analyzing how different emotions are expressed in text. This diversity ensures that the models trained on this dataset can effectively learn to distinguish between the subtle nuances of each emotional class, thereby improving their accuracy and reliability in real-world applications.

## 4. Methodology

**Figure 3:**
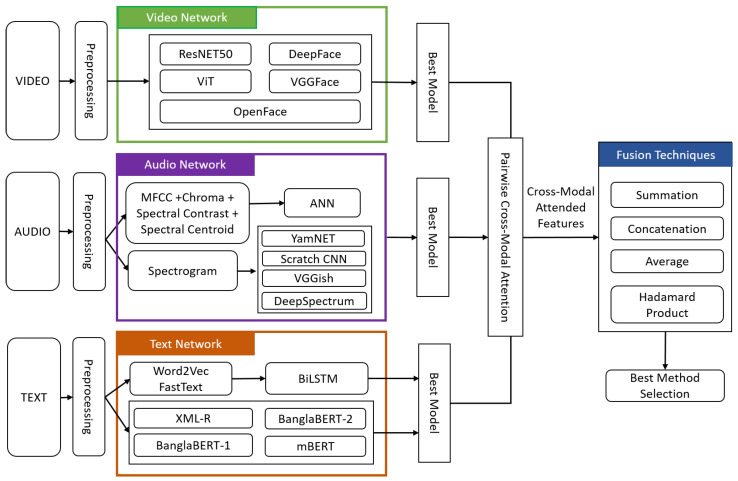
Abstract overview of the multimodal emotion classification system.

Emotions are expressed across multiple channels, including verbal, vocal, and visual modalities, rather than through a single channel. Utilizing various data modes enables a more precise analysis of emotional states, which may be overlooked by unimodal systems. This study combines features from video, audio, and text modalities to make the final prediction for multimodal emotion classification. Figure 3 illustrates the workflow of multimodal emotion classification using the MAViTE-Bangla dataset.

### 4.1. Video Network

The video network is introduced to extract spatial features from the selected frames of the mp4 input video modality. The function of this part of the system is depicted in Figure 3. The steps of video modality processing are represented in this section.

#### 4.1.1. Preprocessing

For the video modality, a uniform approach is taken where five frames are extracted from each video, regardless of the video’s total length. To achieve this, a skip window, denoted as *W*, is defined. This window determines the interval at which frames are sampled from the video. The calculation of *W* is based on the total number of frames *N* in the video and the desired number of frames *L* to be extracted for analysis. The formula used to compute the skip window is provided below:

(1) $$W=max(\left\lfloor \frac{N}{L}\right\rfloor ,1)$$

In this equation, the skip window *W* is determined as the maximum value between the floor division of the total number of frames *N* with the desired sequence length *L* and 1. Subsequently, all frames are resized to (224, 224) to maintain uniform dimensions and reduce computational complexity.

#### 4.1.2. Feature Extraction

Feature extraction transforms raw pixel data from video frames into meaningful representations that capture essential patterns and attributes. Pretrained models such as ResNet-50, ViT, DeepFace, FaceNet, and OpenFace have extracted the features from the selected video frames. Before passing the video frames to these architectures, the frames are passed and resized to (224×224). The deep learning-based feature extraction methods used to extract intricate facial features from video frames are based on CNN. Therefore, they are designed to process images hierarchically, i.e., automatically capturing features at multiple scales and resolutions as the image is passed through the layers [23].

**ResNet-50:** ResNet-50 [24] (residual network with 50 layers) is a robust convolutional neural network (CNN) known for its deep architecture and ability to handle vanishing gradient problems through residual learning. It is widely used for feature extraction due to its strong performance in image recognition tasks. ResNet-50 is pre-trained on the ImageNet dataset [25], a large-scale dataset containing over 14 million images and 1000 object categories, allowing it to learn rich hierarchical features ranging from edges to complex patterns.

**Vision Transformer (ViT):** Vision Transformer (ViT) [26] is a novel model that applies the transformer architecture, initially designed for natural language processing, to image data. ViT divides an image into patches, processes these patches as sequences, and uses self-attention mechanisms to capture long-range dependencies and contextual information. This approach allows ViT to excel at capturing detailed and intricate visual features. ViT is also pre-trained on the ImageNet dataset, leveraging its extensive and diverse image collection to learn effective visual content representations.

**DeepFace:** DeepFace [27] is a deep learning model designed explicitly for facial recognition and analysis. Developed by Facebook, it uses a combination of convolutional neural networks and deep learning techniques to achieve high accuracy in identifying and verifying faces. DeepFace is pre-trained on a large proprietary dataset of over 4 million facial images from more than 4000 persons. This extensive dataset enables DeepFace to extract detailed facial features critical for emotion recognition, such as expressions, landmarks, and subtle variations in appearance. This makes it particularly useful for applications where understanding facial cues is essential.

**FaceNet:** FaceNet [28] is a deep learning model developed by Google for face recognition and clustering. It learns to map face images to a compact Euclidean space where distances reflect face similarity. Trained on large datasets, including CASIA-WebFace and Google’s proprietary datasets with millions of images, FaceNet extracts highly discriminative facial features. This makes face verification, identification, and emotion recognition practical by capturing subtle facial expressions and landmarks variations.

**OpenFace:** OpenFace [29] is an open-source facial behavior analysis model developed by researchers at Carnegie Mellon University. It employs deep learning techniques for facial landmark detection, head pose estimation, and facial action unit recognition. OpenFace is designed to be highly efficient and versatile, making it suitable for real-time applications. It is trained on a diverse set of facial images from various datasets, including the CMU Multi-PIE dataset and the 300 W dataset. This extensive training allows OpenFace to capture and analyze facial features crucial for emotion recognition and human–computer interaction applications.

**EmoAffectNet:** EmoAffectNet [30] is a deep learning model focused on emotion recognition from facial expressions, built on the ResNet-50 architecture. It uses convolutional layers for extracting facial features, followed by fully connected layers for classifying emotions. It incorporates temporal layers like LSTMs or GRUs to analyze video sequences, capturing the dynamic changes in expressions. EmoAffectNet is typically trained on large datasets like AffectNet and FER2013, which are comprehensive but predominantly reflect Western facial expressions and emotions.

**CMT EmoAffectNet:** CMT EmoAffectNet [31] is an advanced version of EmoAffectNet that integrates Contextual and Multitask (CMT) learning. It extends the ResNet-50 backbone by adding multitask learning layers to predict related tasks such as age or gender alongside emotion recognition. However, in our usage, CMT EmoAffectNet has been employed solely for emotion recognition without incorporating external labels like age or gender. The model may include attention mechanisms or contextual layers to enhance emotion detection, particularly in complex scenarios or video data. CMT EmoAffectNet is trained on a combination of emotion datasets like AffectNet and FER2013.

### 4.2. Audio Network

The function of an audio network is to process the audio modality. Here, a WAV file is passed through several steps so that intricate features can be extracted from it.

#### 4.2.1. Preprocessing

For the audio modality, samples are adjusted to a uniform length of 5 s through padding and truncation. Normalization is then performed to achieve consistent volume levels, setting the audio to zero mean and unit variance. The noise reduction technique spectral gating is utilized to reduce background noise and enhance clarity.

#### 4.2.2. Feature Extraction

- **Hand-crafted Feature Extraction:**

Various acoustic features, including Mel-frequency cepstral coefficients (MFCCs), chroma features, spectral centroid, spectral contrast, and spectrograms, are extracted to analyze the audio signal comprehensively. Each feature offers unique insights into different aspects of the audio data.

MFCCs are set to 13 coefficients to capture key characteristics of the audio signal. This is achieved by applying a Mel-scale filter bank to the Fourier transform of the signal. The MFCCs effectively capture the perceptual aspects of sound, making them particularly useful in speech and music analysis [32].

Chroma features represent the energy distribution across the 12 pitch classes of the musical octave. These features are valuable for analyzing the harmonic and melodic content of the audio, helping to identify chords and tonal structures.

The spectral centroid is a measure that indicates the “center of mass” of the audio spectrum. It is associated with the balance of frequencies and is often perceived as the brightness of the sound. Higher values of the spectral centroid correspond to brighter sounds.

Spectral contrast measures the difference between peaks and valleys in the spectrum. This feature captures the variations in the harmonic structure and timbre of the audio, providing insights into the textural differences within the sound.

Spectrograms display the spectrum of frequencies as they vary over time, offering a time–frequency representation of the audio signal. This allows for the observation of how different frequency components evolve, which is crucial for understanding the temporal dynamics of the audio.

To ensure uniformity, these features are truncated and padded to a length of 20, resulting in an 80-feature vector for each audio sample. This standardized vector format allows for consistent comparison and analysis across different audio samples, thereby enhancing the robustness of acoustic analysis in applications such as emotion recognition.

- **Deep Feature Extraction:**

Deep feature extraction involves using advanced deep learning techniques to analyze and enhance handcrafted features further. This process leverages the power of deep neural networks to capture intricate patterns and representations that handcrafted features alone might miss. Therefore, we have utilized a scratch ANN to extract the deep features of MFCC, chroma features, spectral centroid, and spectral contrast. On the other hand, for the spectrogram, we have experimented with several pre-trained deep learning models:

**CNN:** A five-layer convolutional neural network (CNN) is designed to extract features from spectrograms. The network begins with an input layer that accepts spectrogram images of size 224×224 pixels rgb channel. It includes three convolutional layers, each followed by a max pooling layer to reduce the spatial dimensions while capturing essential features. The first convolutional layer uses 64 filters with a 3×3 kernel and the second and third use 128 filters, all activated by the ReLU function. After these convolutional and pooling operations, the data are flattened into a vector. This vector is passed through two fully connected (dense) layers: the first dense layer with 256 neurons activated by ReLU, and the output layer with four neurons activated by softmax, designed for multi-class audio classification. The model is compiled using the Adam optimizer and categorical cross-entropy loss function.

**YamNet:** YamNet [33] is a pre-trained deep learning model developed by Google for audio event detection and classification. It is based on a MobileNetV1 architecture and is trained on the AudioSet dataset, which contains many audio events. YamNet takes audio waveforms as input and outputs class scores for 521 different audio event classes. It is designed to be lightweight and efficient, making it suitable for real-time audio analysis applications.

**VGGish:** VGGish is a Google pretrained deep learning model designed for extracting audio features. It is based on the VGG16 architecture, a convolutional neural network originally designed for image classification. VGGish is adapted for audio by processing log Mel spectrograms of audio clips. The model is trained on a large-scale dataset, including millions of YouTube videos, and provides high-level audio features that can be used for various tasks such as audio classification, event detection, and similarity analysis.

**DeepSpectrum:** DeepSpectrum [34] is a framework that uses pre-trained deep learning models, initially designed for image analysis, to extract features from audio spectrograms. It converts audio signals into visual representations (spectrograms) and then applies image-based deep learning models such as ResNet, Inception, or VGG to these spectrograms. In this work, Inception-ResNet-v2 is chosen for extracting features from spectrograms. DeepSpectrum leverages the power of these image recognition models to capture intricate patterns in the audio data, making it useful for tasks like emotion recognition, sound classification, and other audio analysis applications.

### 4.3. Text Network

The input text data are utilized and encoded to extract deep features.

#### 4.3.1. Preprocessing

In this study, the textual part of the MAViTE-Bangla dataset underwent comprehensive preprocessing to ensure data quality and consistency. The preprocessing steps included tokenization, normalization, and removing stop words and punctuation. Additionally, special characters and numbers were filtered out to enhance the robustness of the subsequent analysis.

#### 4.3.2. Feature Extraction

- **Word Embedding**

We utilized the following word embedding approach followed by the BiLSTM network as a deep feature extractor.

**Word2Vec:** Word2Vec is a well-known and widely adopted word embedding technique used to identify semantic similarities between words within a dataset’s context [35]. Two variants of the Word2Vec algorithms are skip-gram and continuous bag of words (CBOW). According to [36], the skip-gram model performs effectively with small training datasets and accurately represents even rare words or phrases. In this study, Word2Vec is trained using the skip-gram model with a window size of 7, an embedding dimension of 100, and a minimum word count of 4.

**FastText:** The Word2Vec algorithm is limited in handling out-of-vocabulary words, as any word not included in the training set cannot be vectorized with a corresponding embedding. To overcome this limitation, the FastText algorithm was introduced [37]. By leveraging sub-word information, FastText utilizes character n-grams to establish semantic relationships between words within a specific context [38]. This methodology allows for synthesizing embeddings for words not present in the training vocabulary using their constituent n-grams. FastText, like Word2Vec, is available in both skip-gram and CBOW variants. In this study, the FastText algorithm was trained using the skip-gram model with a window size of 5, a character n-gram size of 5, and an embedding dimension of 100.

- **Contextual Embedding**

**mBERT:** We utilized the ’bert-base-multilingual-cased’ model on the MAViTE-Bangla dataset, fine-tuning it by adjusting the batch size, learning rate, and number of epochs. The training of m-BERT [39] included the 104 most widely spoken languages, using the most extensive Wikipedia datasets available, which also covered Bengali. The pre-trained m-BERT model comprises approximately 110 million parameters.

**XLM-R:** XLM-R [40] was trained using a multilingual masked language model. Various innovative training methods improve BERT’s performance, including (1) extending the training duration with more data, (2) using larger batch sizes and longer sequences, and (3) dynamically creating the masking pattern. The XLM-R model significantly outperforms other multilingual BERT models, particularly in low-resource languages. The ‘xlm-Roberta-base’ method is applied to the MAViTE-Bangla dataset with a batch size of 12.

**Bangla BERT:** This study employs two variants of Bangla BERT that are exclusively pre-trained in the Bengali language. The first variant, ‘sagorsarker/Bangla-bert-base’ (referred to as Bangla-BERT-1) [41], is trained on the Bengali corpus from OSCAR (https://oscar-corpus.com/ (accessed on 21 July 2023)) and the Bengali Wikipedia Dump Dataset (https://dumps.wikimedia.org/bnwiki/latest/ (accessed on 21 July 2023)). The second variant, ‘csebuetnlp/banglabert’ (Bangla-BERT-2) [42], is also used. Both pre-trained models are based on the masked language modeling approach described in the original BERT paper [43].

### 4.4. Pairwise Cross-Modal Attention

Cross-modal attention allows the model to focus on the most relevant parts of each modality concerning the other, enhancing the understanding of the combined information. This mechanism has been used in some non-emotion corpuses. For instance, ref. [44] introduces the AVCRFormer model. This novel transformer-based architecture integrates audio and visual features using a spatio-temporal cross-attention fusion strategy for audio–visual speech recognition. This work focuses on a non-emotion corpus, where the cross-attention mechanism is specifically utilized to effectively align and fuse the audio and visual modalities, enhancing the model’s ability to recognize speech in challenging environments. In our case of multimodal emotion corpus, the best models from the video, audio, and text networks are chosen based on their weighted F1-score and prepared for cross-attention between each modality pair. The performance is measured based on the F1-score. Upon extracting features from the three modalities (audio, video, and text), the features of each modality undergo normalization to ensure uniform importance across all features. Following this, a cross-modal attention mechanism is implemented across three modality pairs: audio–video, audio–text, and video–text. In the case of the audio–video pair, this mechanism aids in synchronizing lip movements with speech, enhancing the recognition of spoken emotions. For the audio–text pair, integrating the tone of voice with textual content offers a more nuanced understanding of the sentiment conveyed in the speech. Furthermore, the video–text pair leverages facial expressions and body language to provide additional context to the spoken words, thereby facilitating the disambiguation of textual emotions.

#### 4.4.1. Audio–Video Attention

The audio features are initially projected into a query space using a learned weight matrix. Concurrently, the video features are projected into key and value spaces utilizing distinct learned weight matrices. Critical space is employed for matching with queries, while the value space generates the final attended features. Attention scores are then calculated by taking the dot product of the audio queries and the video keys. Subsequently, these raw attention scores are passed through a softmax function, converting them into probabilities that sum to one. This normalization process aids in determining the relative importance of each video feature concerning each audio feature.

Finally, the normalized attention scores compute a weighted sum of the video values. Thus, an attended audio feature representation is created that emphasizes the most relevant video features. Equations (2)–(7) represent the overall cross-attention mechanism.

(2) $$Q_{A}=W_{Q}^{A}A$$

(3) $$K_{V}=W_{K}^{V}V$$

(4) $$V_{V}=W_{V}^{V}V$$

(5) $$S_{A}V=Q_{A}K_{V}^{T}$$

(6) $$\alpha_{ij}=\frac{exp(S_{AV_{ij}})}{\sum_{k}exp\left(S_{AV_{ik}}\right)}$$

(7) $$A^{\prime }=\sum_{j}\alpha_{ij}V_{V}$$

In the above equations, *A* and *V* represent the audio and video features, respectively. $Q_{A}$ is the query projection of audio features, $K_{V}$ is the key projection of video features, and $V_{V}$ is the value projection of video features. $W_{Q}^{A}$, $W_{K}^{V}$, and $W_{V}^{V}$ are the learned weight matrices for the respective projections. Additionally, $S_{AV}$ represents the attention scores, and $\alpha_{ij}$ is the normalized attention score between the *i*-th audio query and the *j*-th video key. Also, $A^{\prime }$ is the attended audio feature representation based on the video features.

#### 4.4.2. Audio–Text Attention

The same approach has been applied to the audio–text pair. The audio features are projected into a query space using a learned weight matrix. The text features are projected into key and value spaces using different learned weight matrices. Then, attention scores are calculated by taking the dot product of the audio queries with the text keys. After that, the attention scores are passed through a softmax function to obtain normalized probabilities. At last, the normalized attention scores are used to compute a weighted sum of the text values, resulting in an attended audio feature representation based on text features. Equations (8)–(13) illustrates the mechanism of audio–text cross-attention:

(8) $$Q_{A}=W_{Q}^{A}A$$

(9) $$K_{T}=W_{K}^{T}T$$

(10) $$V_{T}=W_{V}^{T}T$$

(11) $$S_{A}T=Q_{A}K_{T}^{T}$$

(12) $$\beta_{ij}=\frac{exp(S_{AT_{ij}})}{\sum_{k}exp\left(S_{AV_{ik}}\right)}$$

(13) $$A^{\prime \prime }=\sum_{j}\beta_{ij}V_{T}$$

In the above equations, *A* and *T* represent audio and text features, respectively. $Q_{A}$ is the query projection of audio features, $K_{T}$ is the key projection, and $V_{T}$ is the value projection of text features. $W_{Q}^{A}$, $W_{K}^{T}$, and $W_{V}^{T}$ are the learned weight matrices for the respective projections. Additionally, $S_{AT}$ represents the attention scores, and βij is the normalized attention score between the *i*-th audio and *j*-th text features. Also, $A^{\prime \prime }$ is the attended audio feature representation based on the text features.

#### 4.4.3. Video–Text Attention

The video features are projected into a query space using a learned weight matrix. The text features are projected into key and value spaces using different learned weight matrices. Attention scores are calculated by taking the dot product of the video queries with the text keys. The attention scores are passed through a softmax function. The normalized attention scores are used to compute a weighted sum of the text values, resulting in an attended video feature representation based on text features. The mechanism of this cross-attention can be observed in Equations (14)–(16).

(14) $$S_{V}T={\left(W_{V}V\right)}^{T}W_{T}^{T}$$

(15) $$\gamma_{ij}=\frac{exp(S_{VT_{ij}})}{\sum_{k}exp\left(S_{VT_{ik}}\right)}$$

(16) $$V^{\prime }=\sum_{j}\gamma_{ij}V_{T}$$

where $W_{V}$ and $W_{T}$ are the learned weight matrices for the respective projections. Additionally, $S_{VT}$ represents the attention scores, and βij is the normalized attention score between the *i*-th audio and *j*-th text features. Also, $V^{\prime }$ is the attended audio feature representation based on the text features.

### 4.5. Fusion Methods

After obtaining the cross-modal features from each pair, we combined the three pairs using different fusion methods. The best fusion method was selected based on their weighted F1-score, and we proceeded to further experimentation.

#### 4.5.1. Summation Fusion

Summation fusion combines features from multiple modalities by summing their respective values element-wise. Given feature vectors $f_{1},f_{2},\ldots ,f_{n}$ from *n* modalities, the fused feature vector $f_{\mathrm{sum}}$ is computed as follows:

$$f_{\mathrm{sum}}=f_{1}+f_{2}+\cdots +f_{n}$$

This method ensures that contributions from each modality are equally weighted, resulting in a single fused representation.

#### 4.5.2. Concatenation

Concatenation merges features from different modalities by joining them end-to-end to form a single extended feature vector. Given feature vectors $f_{1},f_{2},\ldots ,f_{n}$ from *n* modalities, the fused feature vector $f_{\mathrm{concat}}$ is computed as follows:

$$f_{\mathrm{concat}}=\left[f_{1};f_{2};\cdots ;f_{n}\right]$$

where [;] denotes the concatenation operation. This approach preserves the original dimensionality of each modality’s features.

#### 4.5.3. Average Fusion

Average fusion combines features by calculating the mean value of the corresponding features across modalities. Given feature vectors $f_{1},f_{2},\ldots ,f_{n}$ from *n* modalities, the fused feature vector $f_{\mathrm{avg}}$ is computed as follows:

$$f_{\mathrm{avg}}=\frac{1}{n}\sum_{i=1}^{n}f_{i}$$

This method helps balance the contributions from each modality, providing a more stable and generalized representation.

#### 4.5.4. Hadamard Product Fusion

Hadamard product fusion integrates features by performing element-wise multiplication of the corresponding features from different modalities. Given feature vectors $f_{1},f_{2},\ldots ,f_{n}$ from *n* modalities, the fused feature vector $f_{\mathrm{had}}$ is computed as follows:

$$f_{\mathrm{had}}=f_{1}⊙f_{2}⊙\cdots ⊙f_{n}$$

where ⊙ denotes the element-wise multiplication. This approach emphasizes the interactions and commonalities among the features of different modalities.

### 4.6. Proposed Method

**Figure 4:**
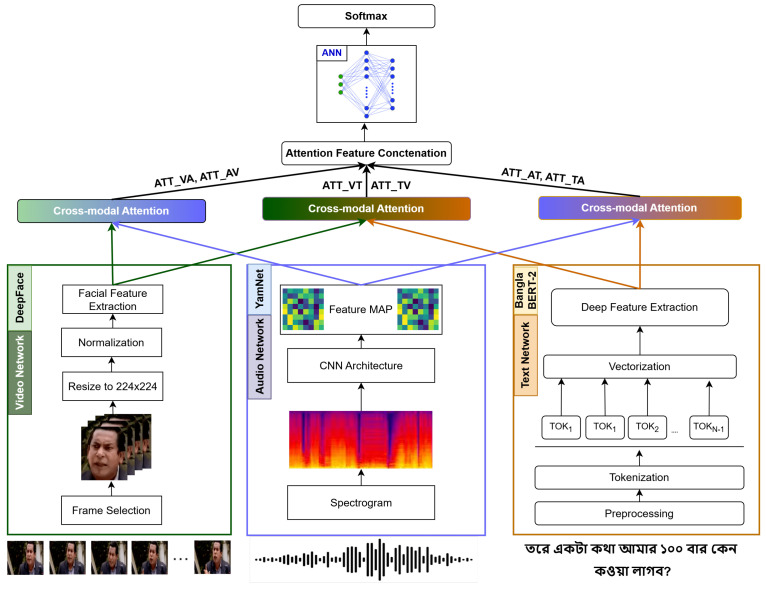
Proposed MEC (Multimodal Emotion Classification) architecture using cross-modal attention.

The proposed architecture is illustrated in Figure 4. We employed two distinct forms of cross-attention mechanisms to facilitate the interaction between features from each modality. The first form, which is already mentioned, includes audio–video (Att_AV), audio–text (Att_AT), and video–text attention (Att_VT), and the second form involves video–audio (Att_VA), text–audio (Att_TA), and text–video attention (Att_TV). In the second form, video features act as queries to attend to audio features in the video–audio attention mechanism. In contrast, text features serve as queries for audio and video features in the text–audio and text–video attention mechanisms.

The proposed method combines these two cross-attention mechanisms to enhance the interaction between modalities comprehensively. Doing so ensures that each modality effectively informs and enriches the others, capturing a more holistic view of the multimodal data. Concatenation emerged as the most effective method for the fusion of these cross-attended features. This approach combines the enriched features from each modality into a single, comprehensive feature vector, facilitating robust and more accurate multimodal emotion recognition.

## 5. Experiments

This section provides a concise summary of the experimental results. The metrics evaluated include precision (Pr.), recall (Re.), F1-score (F1.), and accuracy (Acc.), both for single modalities and multimodality. The model with the highest F1-score was selected as the best-performing model. Table 5 presents the unimodal results for various approaches across the audio, video, and text modalities.

### 5.1. Unimodal

Table 5 shows the performance of unimodal approaches on audio, video, and text modalities.

#### 5.1.1. Audio Modality

We assessed five methods for the audio modality. A1 uses statistical features: MFCC + Chroma + Spectral Contrast + Spectral Centroid using an artificial neural network (ANN) and achieved an F1-score of 0.59. A spectrogram is utilized in A2, A3, A4, and A5. Among them, A3 and YamNet performed better, with a 0.62 F1-score. Scratch CNN performed lowest as it is not a pre-trained model.

#### 5.1.2. Video Modality

The video modality was evaluated using inputs of five and ten frames across five models: ResNet-50, DeepFace, Vision Transformer (ViT), FaceNet, OpenFace, EmoAffectNet, and C MT EmoAffectNet. The DeepFace model consistently outperformed the others. For the five-frame input (Method V2), DeepFace achieved the highest F1-score of 0.71. Similarly, the ten-frame input (Method V7) maintained a high F1-score of 0.70. In contrast, the ResNet-50 and ViT models exhibited moderate performance, with ResNet-50 achieving F1-scores of 0.65 (Method V1) for five frames and 0.62 (Method V6) for ten frames, and ViT attaining F1-scores of 0.65 (Method V3) for five frames and 0.64 (Method V8) for ten frames. On the other hand, FaceNet and OpenFace showed comparative performance. Moreover, EmoAffectNet and C MT EmoAffectNet showed decent performance, but they struggled to accurately detect subtle emotions within the Bangla cultural context. Interestingly, DeepFace, with five frames, outperformed its ten-frame counterpart, indicating that five frames are sufficient for more effective emotion recognition.

#### 5.1.3. Text Modality

The evaluated text modality methods based on Word2Vec, FastText, and transformer-based models. Bangla BERT-2 (Method T6) achieved the highest F1-score of 0.76, demonstrating its strong ability to capture emotional nuances in text data. XML-R (Method T4) also showed commendable performance with an F1-score of 0.70. In contrast, BiLSTM models utilizing Word2Vec (Method T1) and FastText (Method T2) displayed lower performance, with F1-scores of 0.51 and 0.52, respectively.

### 5.2. Multimodal

The multimodal approach for emotion recognition leverages the best-performing model from each modality: A3 for audio, V2 for video, and T6 for textual feature extraction. We implemented a pair-wise cross-modal attention mechanism encompassing six cross-attention feature pairs, i.e., audio–video (Att_AV), audio–text (Att_AT), video–text (Att_VT), video–audio (Att_VA), text–audio (Att_TA), and text–video (Att_TV). Various fusion techniques were explored to combine the cross-modal attended features, and a performance comparison of these strategies is illustrated in Table 6. It is evident from the table that the concatenation method outperforms the others, achieving an F1-score of 0.64.

This superior performance is attributed to the method’s ability to preserve each modality’s full diversity of features by merging them into a single comprehensive feature vector. This approach retains the unique characteristics of each modality, which is essential for multimodal data, as different modalities can provide different information.

**Figure 5:**
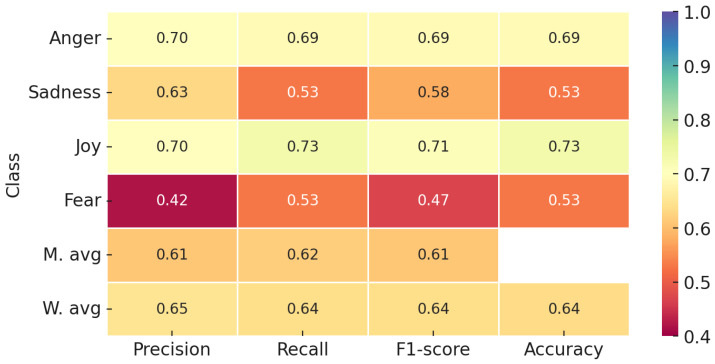
Classification report of the proposed approach.

Figure 5 illustrates the class-wise performance metrics of the proposed multimodal approach. The joy class achieved the highest F1-score of 0.71 and recall of 0.73, demonstrating a solid capability to recognize joyful expressions. The anger class exhibited the highest precision at 0.70, with a balanced performance and an F1-score of 0.69. Although the fear class had a precision of 0.42, it showed a recall of 0.53, indicating that while it missed some instances, it accurately identified fear in many cases. Conversely, the sadness class had relatively lower performance, with an F1-score of 0.58, underscoring the challenges in accurately detecting sadness.

Table 7 shows some test samples and comparisons of the unimodal and multimodal prediction results. The video frames and texts can be understood from the table, although the audio content cannot be fully understood. We can see that in Example 1, the video modality is predicted as joy and audio and text are predicted as sadness, which also matches the multimodal prediction and original label. We can see a man smiling at the frames, which drives the video model to predict it as Joy. However, if we see the text, we can realize it represents some pitiful situation, and the tone represents the same. In Example 2, the text is predicted as anger, whereas audio and video are joy. The content of the text suggests an annoying situation, but the tone includes laughs, and so do the video frames. In Example 3, something different happened: primary modalities, audio and video predict joy, which is not the original label. The original label matched the text model’s prediction. As all of the above examples are labeled with the overall understanding of the content, the cross-modal features were mapped so that the multimodal prediction can extract the correct emotion level.

### 5.3. Ablation Study

To show the effectiveness of our method, we reduced the cross-attention pairs. We made two groups: (1) audio–video (Att_AV), audio–text (Att_AT), and video–text (Att_VT) cross-attention pairs and (2) another is the reverse, i.e., video–audio (Att_VA), text–audio (Att_TA), and text–video (Att_TV) cross-attention pairs. The results are presented in Table 8. We can see that using six pairs of cross-attended features enhances the F1 score by 0.2. This increases the interaction between modalities leading to improved emotion recognition accuracy. This ablation study represents the importance of inter-pair dependencies. For instance, video–audio cross-attended features alone cannot improve the system but adding audio–video cross-attended features can improve the overall performance.

### 5.4. Comparison with Existing Work

There are relatively very few studies conducted with three modalities of emotion. Table 9 compares the proposed model and two existing multimodal emotion recognition models. Notably, the proposed model achieves a superior F1-score. The model in [16] records the lowest F1-score of 0.57, which can be attributed to an excessive reduction in handcrafted audio features, resulting in the loss of vital information essential for precise emotion recognition.

### 5.5. Error Analysis

**Figure 6:**
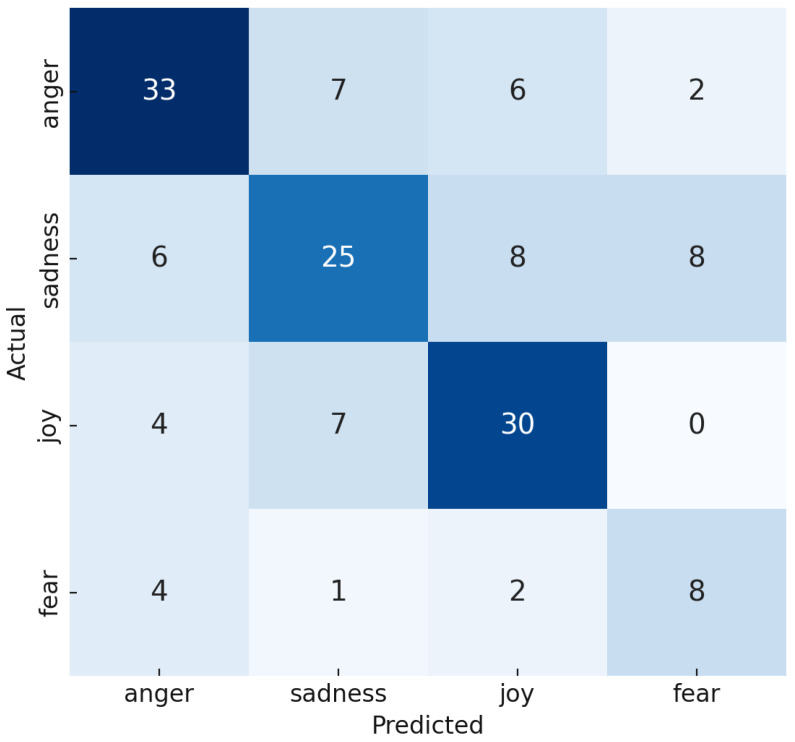
Confusion matrix of the test set prediction. The different colors in the confusion matrix represent the intensity of correct and incorrect predictions, with darker colors indicating higher values and lighter colors indicating lower values.

The confusion matrix depicted in Figure 6 reveals a significant misclassification pattern between the emotions of sadness and anger. Specifically, six instances of sadness were incorrectly classified as anger. We also calculated the Jaccard similarity to find inter-class similarities for the textual data shown in Table 10, and it shows a higher similarity of 0.55 between anger and sadness. The misclassification can be attributed to the acoustic similarities between these two emotions. Both high-pitched shouting, characteristic of anger, and the crying associated with sadness share similar tonal qualities, leading to confusion for the model.

**Figure 7:**
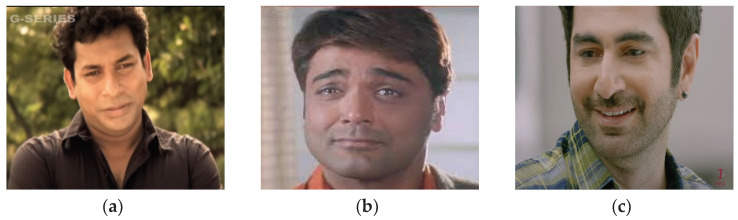
Facial expression with smile exhibiting sad emotion. Here, (**a**–**c**) shows smiling faces but the corresponding multimodal data are labeled with sadness.

Additionally, eight instances of sadness were misclassified as fear. This misclassification might stem from the overlapping vocal expressions and physiological responses between sadness and fear, such as quivering voices and hesitations, making it challenging for the model to differentiate between these emotions accurately. Likewise, eight instances of sadness were misclassified as joy, with a higher Jaccard similarity of 0.52 (Table 10). Also, sadness and joy can sometimes exhibit subtle facial similarities. For instance, a slight smile, which could be subdued or resigned in sadness, might be misinterpreted by the model as a joy class. Some images of this type of sample are presented in Figure 7.

Furthermore, the fear class exhibits a high rate of misclassification, with four samples erroneously identified as anger, another two as joy, and one as sadness. This issue is primarily due to the relatively small size of the fear class within the dataset, which hinders the model’s capacity to learn distinguishing features effectively. The insufficient data for fear limit the model’s exposure to diverse expressions of this emotion, impairing its performance. Enhancing the dataset size for underrepresented classes and improving the model’s capability to discern subtle emotional expressions can significantly boost the overall performance of the emotion recognition system.

## 6. Discussion

This work introduces a pioneering approach to classify multimodal emotions in Bengali, leveraging audio, video, and text modalities in a novel way. While our dataset, comprising 1002 samples, may seem modest compared to those in other languages, we have prioritized quality over quantity. As the first step in the field of multimodal emotion in Bengali, we believe our work will inspire further research and the creation of larger, more diverse datasets in the future. Additionally, the dataset has a class imbalance, especially in the ‘fear’ category, which affects the model’s performance and biases it towards more represented emotions. Here, we wanted to select fear samples that rigorously represented fear, avoiding mixed emotions to ensure high-quality annotations. We plan to expand the dataset on underrepresented emotions in future work. Moreover, we aim to add more classes and more data to the existing classes in our future work. Also, the sources of data collection, i.e., movies, dramas, and vlogs, may only partially encompass the range of real-world situations where emotion recognition is critical. These sources were chosen for their accessibility and the variety of emotional expressions they provide. However, we recognize the importance of diverse real-world scenarios and plan to expand the dataset to include more varied and spontaneous contexts in future work. While this work is a primary attempt in Bengali to classify multimodal emotion constituting audio, video, and text modalities, we hope to apply an English or other language-based multimodal emotion dataset to the proposed method to further demonstrate its universality and efficacy.

## 7. Conclusions

This study introduced a cross-modal fusion framework to classify multimodal emotions into four categories: anger, fear, joy, and sadness. The framework addresses the complexities of emotion recognition across different modalities by integrating Bengali acoustic, visual, and textual features. To facilitate the evaluation of the proposed framework, a novel Bengali multimodal dataset named MAViTE-Bangla has been created, comprising 1002 samples. Experimental outcomes demonstrated that the combination of different feature extraction techniques, such as YamNet (acoustic), DeepFace (visual), and Bangla BERT-2 (textual) with ANN classifier outperformed other exploited techniques, achieving the highest F1-score (0.64) for MEC. Despite the current multimodal approach yielding a lower F1-score than unimodal results, the advantages of multimodality, such as providing a more comprehensive, robust, and context-aware understanding of emotions, underscores its value. These benefits justify the ongoing use and further development of multimodal systems. By emphasizing the broader perspective and the potential for future enhancements, we can bolster the adoption of AVaTER in emotion recognition tasks, paving the way for more accurate and nuanced emotion detection systems.

## Figures and Tables

**Table 1:** Data distribution among classes.

Class	Train	Validation	Test
Anger	221	48	48
Fear	70	15	15
Joy	191	41	41
Sadness	218	47	47
Total	700	151	151

**Table 2:** Statistics for audio data.

Class	Min Duration (s)	Max Duration (s)	Mean Duration (s)	Total Duration (s)
Anger	1.022	7.002	3.210	950.071
Fear	2.001	14.008	5.491	549.061
Joy	0.952	9.015	2.937	757.709
Sadness	1.207	7.012	3.391	986.822

**Table 3:** Statistics for video data.

Class	Max Frame Rate (fps)	Min Frame Rate (fps)	Max Resolution	Min Resolution	Max File Size (KB)	Min File Size (KB)
Anger	30.0	24.00	1920×1080	208×210	10,075.15	141.32
Fear	30.0	23.98	2560×1440	450×360	6845.49	99.54
Joy	30.0	23.98	1920×1080	206×174	9975.87	102.99
Sadness	30.0	24.00	1920×1080	176×162	11,053.24	91.93

**Table 4:** Statistics for text data.

Class	Total Words	Total Sentences	Average Word Length	Average Sentence Length	Lexical Diversity
Anger	2395	88	4.356	27.216	0.509
Fear	996	34	4.070	29.294	0.495
Joy	1819	54	4.318	33.685	0.537
Sadness	1971	31	4.337	63.581	0.464

**Table 5:** Unimodal results for audio, video, and text modalities.

Modality	Approach/ Features	Model	Method No.	Pr	Re	F1	Acc
Audio	MFCC + Chroma+ Spectral Contrast + Spectral Centroid	ANN	A1	0.59	0.59	0.59	0.59
	Spectrogram	CNN	A2	0.57	0.55	0.55	0.56
		**YamNet**	**A3**	**0.63**	**0.62**	**0.62**	**0.62**
		Vggish	A4	0.59	0.59	0.60	0.59
		DeepSpectrum	A5	0.61	0.60	0.59	0.61
Video	5 Frames	ResNet-50	V1	0.65	0.66	0.65	0.65
		**DeepFace**	**V2**	**0.74**	**0.72**	**0.71**	**0.72**
		Vit	V3	0.67	0.66	0.65	0.67
		FaceNet	V4	0.69	0.67	0.66	0.67
		EmoAffectNet	V5	0.65	0.66	0.68	0.68
		C MT EmoAffectNet	V6	0.66	0.67	0.67	0.69
		OpenFace	V7	0.72	0.69	0.67	0.69
	10 Frames	ResNet-50	V8	0.64	0.63	0.62	0.63
		DeepFace	V9	0.73	0.71	0.70	0.71
		Vit	V10	0.66	0.65	0.64	0.66
		FaceNet	V11	0.68	0.67	0.65	0.66
		EmoAffectNet	V12	0.65	0.66	0.66	0.67
		C MT EmoAffectNet	V13	0.66	0.66	0.65	0.67
		OpenFace	V14	0.71	0.69	0.70	0.70
Text	Word2Vec	BiLSTM	T1	0.52	0.50	0.50	0.52
	FastText	BiLSTM	T2	0.53	0.52	0.52	0.52
	Transformer	MBERT	T3	0.53	0.52	0.53	0.52
		XML-R	T4	0.68	0.67	0.65	0.66
		Bangla BERT-1	T5	0.76	0.76	0.76	0.76
		**Bangla BERT-2**	**T6**	**0.77**	**0.76**	**0.76**	**0.76**

*Note:* The bold texts in table cells indicate high performing model names and their scores.

**Table 6:** Fusion techniques for cross-modal attended features.

Fusion Techniques of Cross-Modal Attended Features	Pr	Re	F1	Acc
Summation	0.60	0.59	0.59	0.60
Averaging	0.56	0.57	0.55	0.56
Concatenation	0.65	0.64	0.64	0.64
Hadamard Product	0.62	0.61	0.62	0.63

**Table 7:** Example with comparison between unimodal and multimodal approaches.

No.	Modality	Content	Unimodal Prediction	Multimodal Prediction	Original Label
1	Video	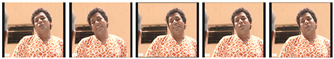	Joy	Sadness	Sadness
	Audio	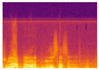	Sadness		
	Text	আসলে আপনি আমাকে ভুল বুঝতেছেন। (Actually you are getting me wrong.)	Sadness		
2	Video	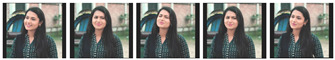	Joy	Joy	Joy
	Audio	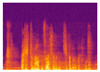	Joy		
	Text	আচ্ছা তুমি এরকম ফাজলামি করতেছ কেন হঠাৎ করে! (Why are you doing such nonsense all of a sudden!)	Anger		
3	Video	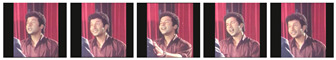	Joy	Anger	Anger
	Audio	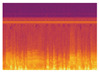	Joy		
	Text	আচ্ছা বলুন তো আপনার পূর্বপুরুষেরা কি খানসামা ছিলেন? (So tell me, were your ancestors housekeepers?)	Anger		

**Table 8:** Cross-modal attention strategies and their corresponding performance metrics.

Crossmodal Attention Strategies	Pr.	Re.	F1.	Acc.
Att_AV + Att_VT + Att_AT	0.63	0.63	0.62	0.63
Att_VA + Att_TV + Att_TA	0.62	0.61	0.62	0.62
Att_AV + Att_VT + Att_AT + Att_VA + Att_TV + Att_TA	0.65	0.64	0.64	0.64

**Table 9:** Performance Comparison of Proposed Model with Existing Approaches.

Multimodal Feature Extraction	Classifier	F1
Wav2vec2.0 + videoMAE + BERT [17]	SVM	0.59
(Handcrafted audio features + CNN-LSTM) + Inception-ResNet-v2 + Word2Vec [16]	ANN	0.57
Convolutional Deep Belief Network (CDBN) [45]	SVM	0.58
Bidirectional LSTM + Attention-based Fusion [46]	Softmax	0.61
YamNet + DeepFace + Bangla BERT-2 (Proposed)	ANN	0.64

**Table 10:** Jaccard similarity index between emotion classes.

Class	Anger	Sadness	Joy	Fear
Anger	1.00	0.55	0.48	0.40
Sadness	-	1.00	0.52	0.45
Joy	-	-	1.00	0.42
Fear	-	-	-	1.00

## Data Availability

Data are contained within the article.
